# [Retracted] Combination of HSP90 and autophagy inhibitors promotes hepatocellular carcinoma apoptosis following incomplete thermal ablation

**DOI:** 10.3892/mmr.2026.13838

**Published:** 2026-03-05

**Authors:** Fen Chen, Haiyang Xie, Haiwei Bao, Laurencia Violetta, Shusen Zheng

Mol Med Rep 22: 337–343, 2020; DOI: 10.3892/mmr.2020.11080

Following the publication of the above paper, a concerned reader drew to the Editor's attention that, concerning the western blots shown in Fig. 2 on p. 340, the protein bands representing the LC3 II data in the two left-hand lanes of Fig. 2A were strikingly similar to the two blots representing the P62 data in the left-hand lanes of the gel slice shown in Fig. 2C, albeit the bands appeared to have been horizontally flipped, with horizontal and vertical resizing.

After assessing this issue in the Editorial Office, the Editor of *Molecular Medicine Reports* has determined that the concerns of the reader were well founded; therefore, this paper has been retracted from the Journal on account of a lack of confidence in the authenticity of the presented data. The authors were asked for an explanation to account for these concerns, but the Editorial Office did not receive a satisfactory reply. The Editor regrets any inconvenience that may have been caused to the readership of the Journal.

